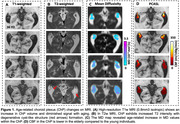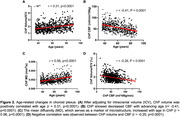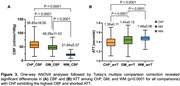# Age‐Related Structural and Vascular Changes in the Choroid Plexus: Insights from the HCP Dataset

**DOI:** 10.1002/alz.093708

**Published:** 2025-01-09

**Authors:** Zhe Sun, Chenyang Li, Thomas Wisniewski, Yulin Ge

**Affiliations:** ^1^ NYU Grossman School of Medicine, New York, NY USA; ^2^ Alzheimer's Disease Research Center, New York University Langone Health, New York, NY USA

## Abstract

**Background:**

The choroid plexus (ChP) plays a vital role in CSF production and waste clearance. While existing imaging studies have established connections between ChP volume changes and age‐related neurodegenerative diseases, a comprehensive investigation into the microstructural and vascular changes associated with aging remains insufficient. This study aims to explore ChP changes in normal aging using diffusion and perfusion MRI in the HCP‐Aging dataset to enhance our understanding of age‐related microstructural and vascular changes in the ChP.

**Method:**

Our analysis included 641 healthy participants (age: 36‐90 years; mean±SD = 60±16 years; 282 males) with the following MRI parameters: (1) T1 MPRAGE for ChP segmentation[1]; (2) Diffusion MRI for quantification of mean diffusivity (MD); (3) PCASL MRI for quantification of cerebral blood flow (CBF) and arterial transit time (ATT). The ChP mask was co‐registered with MD, CBF, and ATT maps to extract corresponding values. Pearson's correlation and one‐way ANOVA were used for age effects and perfusion property comparisons among gray matter (GM), white matter (WM), and ChP, respectively.

**Result:**

ChP imaging revealed increased volume, increased MD, and decreased CBF across different age groups (Figure 1). Figure 2 demonstrated a positive correlation between ChP volume and age (r = 0.31, p<0.0001), while ChP CBF declined with age (r = ‐0.41, p<0.0001) and MD positively correlated with age (r = 0.56, p<0.0001). Adjusting for age and sex, ChP CBF negatively correlated with volume (r = ‐0.26, p < 0.0001). One‐way ANOVA analyses indicated significant differences in CBF and ATT among ChP, GM, and WM (p<0.0001 for all comparisons), with ChP exihbiting the highest CBF and shortest ATT (Figure 3).

**Conclusion:**

In addition to volume changes, we found significant age‐related diffusion and perfusion changes in the ChP in this large dataset. The decline in CBF and the increase in MD with aging suggest vascular degeneration and compensatory dystrophic hyperplasia of surrounding tissues of ChP. These findings in normal aging set a crucial normative reference for future studies on cognitive impairment in abnormal aging processes References: 1. Tadayon, E., et al. J Alzheimers Dis (2020).